# Exploring the experiences of wellbeing, health, and healthcare among women who have been domestically sex trafficked in Ontario, Canada: A qualitative study protocol

**DOI:** 10.1371/journal.pone.0299500

**Published:** 2024-03-06

**Authors:** Rhonelle Bruder, Robin Mason, Charmaine C. Williams, Janice Du Mont

**Affiliations:** 1 Women’s College Research Institute, Women’s College Hospital, Toronto, Canada; 2 Dalla Lana School of Public Health, University of Toronto, Toronto, Canada; 3 Factor-Inwentash Faculty of Social Work, University of Toronto, Toronto, Canada; McGill University, CANADA

## Abstract

**Introduction:**

Although there is a growing body of evidence to suggest that persons who have been sex trafficked can suffer devastating health consequences and often face challenges accessing suitable care that addresses their health and overall well-being, little existing research has adopted a survivor-informed approach. Centering the voices of sex-trafficked women in this research will provide valuable insights into their health-related experiences and can help lay the foundation for survivor-centric healthcare responses.

**Methods and analysis:**

Using a semi-structured interview guide, we will interview women who have been domestically sex trafficked in Ontario; recruitment will continue until data saturation is reached. Interview questions and prompts will elicit information about women’s experiences prior to, during, and after their trafficking ordeal, with particular attention paid to their encounters with healthcare providers. Intersectionality theory will inform strategies for recruitment, data collection, and data analysis. Data will be analyzed deductively as well as inductively using Braun and Clarke’s six phases of reflexive thematic analysis. The study’s design was informed by the consolidated criteria for reporting qualitative research (COREQ), which ensures a comprehensive and robust reporting of interview data. We will continue to adhere to the COREQ checklist throughout the data collection, analysis, and findings write-up phases, helping to ensure methodological accuracy and transparency.

**Discussion:**

To our knowledge, this will be the first Canada-specific investigation to apply intersectionality theory to explore the experiences of well-being, health, and healthcare from the perspectives of women who have been domestically sex trafficked. The results of this study hold the potential to improve responses to trafficking within the healthcare sector. Specifically, the findings could be used to inform the development of education materials and curricula for medical students and continuing professional education for health and allied healthcare providers. They could also inform the creation of patient experience surveys and intake forms for sex trafficked patients.

## Introduction

Sex trafficking, a severe violation of human rights and detriment to health equity worldwide, involves the recruitment, transportation, or holding of victims for the purpose of sexual exploitation [1]. When the entirety of the crime happens within a single country, it is referred to as domestic sex trafficking [2]. In Canada, there were 3,541 reported incidents of human trafficking between 2011 and 2021, with 2,688 victims identified [3]. Among these cases, 96% involved women and girls, with approximately 69% of these victims under the age of 25 [3,4]. Indigenous women and girls, migrants and recent immigrants, 2SLGBTQI+ persons, children and youth enmeshed in the child welfare system, and those who are socio-economically disadvantaged have been identified as most vulnerable [1]. While the province of Ontario has been noted as a prime site for domestic sex trafficking, accounting for 62% of reported incidents [3,5], the actual scope and scale of human trafficking in Ontario and throughout Canada is difficult to estimate. This difficulty is partly due to the limitations of formal data collection tools; the crime’s clandestine and secretive nature; under-reporting by victims due to fear of retribution; stigma and mistrust of authorities; and under-recognition by service providers due to inadequate training and lack of knowledge [6–12]. Another limitation is that current datasets fail to distinguish between sex trafficking and other forms of human trafficking [13].

Being sex trafficked can have devastating health consequences for women, such as post-traumatic stress disorder, depression, suicidal ideation, increased rates of substance use, and sexually transmitted infections [14–18]. However, the body of research on sex trafficking in Canada remains sparse, particularly from a healthcare standpoint, despite evidence identifying health services as an important access point for those seeking help [13,19,20]. International scholarly research indicates that survivors of trafficking who present to healthcare facilities are often reluctant to disclose their situation due to actual or perceived stigmatization for health concerns for which they are seeking care (e.g. sexually transmitted infections, unintended pregnancy, psychological distress) or perceived immoral behaviours (e.g. substance use, sex work, homelessness) [21–23]. Further, in the absence of disclosure, providers have said they are unable to recognize indicators of sex trafficking and, therefore, may fail to appropriately intervene or care for this population [24–27]. Learning from survivors’ experiences could address many of these gaps and provide a wealth of information needed to improve providers’ recognition and treatment of trafficked persons.

There is little published academic literature on sex trafficking from the perspectives of sex-trafficked women. Within the healthcare context, this is particularly evident. A handful of studies have examined sex-trafficked adults’ interactions within the healthcare system [28–30] and individual health experiences [31,32]. One mixed-methods Delphi study also investigated survivor-recommended strategies for training healthcare providers [19]. The findings of this study underscored the importance of healthcare providers using trauma-informed and rights-based approaches to improve the healthcare experiences of trafficked persons.

Additionally, one study specific to the Canadian context examined the healthcare experiences of survivors of trafficking residing in Montreal, Quebec [33] and emphasized the necessity for multidisciplinary community-based interventions to enhance care coordination and continuity.

Recommendations from across the broader literature have highlighted the pivotal role of listening to survivors’ voices in all aspects of trafficking identification and response efforts [34,35]. Therefore, our study will explore the experiences of women who have been domestically sex trafficked in Ontario, Canada, focusing on their well-being, health, and encounters with healthcare providers. By highlighting their perspectives and voices, we aim to gain insight into the diverse strategies survivors have devised to manage health and well-being, access healthcare services, and interact with healthcare providers. The findings of this study have the potential to lay the foundation for developing health services that are better suited to addressing the unique needs of sex-trafficked individuals.

## Materials and methods

### Study design

In this proposed exploratory qualitative study, we will complete one-on-one interviews to develop deep understanding and create detailed descriptions of domestically sex-trafficked women’s individual health and healthcare experiences [36]. Qualitative methods are well-suited for exploring individuals’ personal histories, perspectives, and experiences, particularly when examining sensitive topics [37]. Intersectionality theory will be applied to highlight the diverse lived experiences of women who have been domestically sex trafficked. Intersectionality is a theoretical framework coined by legal scholar Kimberlé Crenshaw, which examines how a person’s social and political identities intersect to create different modes of discrimination and privilege [38]. Intersectionality provides a novel theoretical approach to the critical examination of the sex trafficking of women, challenging the idea of homogeneity within experiences. Instead, intersectionality highlights the significance of socio-structural inequalities and power dynamics stemming from social identities that can increase vulnerability to exploitation, impact the ability to access services, and the quality of the care provided. The consolidated criteria for reporting qualitative research (COREQ) [39] was used in the development of this study protocol [S1 Checklist].

### Community engagement

We will apply a community-engaged (CE) approach to this study, which involves the “process of working collaboratively with and through groups of people affiliated by geographic proximity, special interest, or similar situations to address issues affecting the well-being of those people” [40, p.9]. The benefits of CE research in the planned study include incorporating insights from those with lived experience into the research, conducting research that can have a real-life application to improving services for those impacted in the community, designing more culturally appropriate interventions, and increasing study recruitment through accessing community networks [41]. We will establish a community advisory group (CAG) consisting of three to four women who have previously experienced sex trafficking in Ontario and two to three healthcare providers practicing within the same jurisdiction. The recruitment of CAG members with lived experiences will be achieved through outreach to community organizations that provide services or support to sex-trafficked women. In parallel, healthcare providers with prior experience caring for sex-trafficked patients will be recruited via established networks within the research team. The CAG will include women and providers from varied racial and ethnic backgrounds. The CAG will meet with the research team every two months over the course of the study to help inform the research design and implementation, provide feedback on the interview guide and recruitment strategies, aid in the interpretation of preliminary findings and themes, and develop and share knowledge translation materials. Members with lived experience will receive an honorarium for their contributions to the study.

### Researching vulnerable populations

The study will investigate the experiences of women who have been domestically sex trafficked in Ontario, Canada. Though an admittedly vulnerable population, we recognize and will acknowledge the resilience of those who have been sex trafficked. There is a growing body of literature that has identified the therapeutic benefits of sharing stories of physical and sexual violence through research interviews. These benefits include increased well-being and healing and decreased experiences of isolation and stigma [42–45]. Given the sensitive nature of the topic, this study will adhere to the ethical guidelines as outlined in the World Health Organization’s Ten Guiding Principles for Ethical and Safe Interviewing of Trafficked Women [46]. Additionally, the study plan incorporates supplementary measures to ensure the safety and well-being of the participants. These include asking participants to confirm that they are situated in a secure and private environment before commencing the interview. Further, regular check-ins will be carried out throughout the interview process to ensure the comfort of participants. Lastly, transparent details regarding the withdrawal process will be provided, allowing participants the option to withdraw their data up until the completion of the analysis. These measures aim to safeguard the participants’ well-being and uphold the research’s ethical integrity.

### Recruitment

We will recruit domestically sex-trafficked women in Ontario, Canada. To be eligible for the study, participants will need to identify as cis or transgender women residing in Ontario, Canada, have the ability to speak and read in English, provide informed consent, and be 18 years or older at the time of the interview. Additionally, to minimize the potential of re-traumatization, participants will need to be at least six months post-trafficking [47]. Recruitment, which is expected to begin in early 2024, will involve purposive sampling to enroll approximately 15 participants or until data saturation is achieved [48,49]. Saturation will be deemed to have been achieved when no new information emerges from the interviews [50]. Diverse strategies will be employed for participant recruitment, including contacting organizations that provide care to sex-trafficked persons; using the research team’s professional and personal networks; and social media platforms such as Instagram, LinkedIn, and Twitter. Recruitment via social media will direct interested individuals to a research team member for more information. Detailed discussions about study participation will not take place on any social media platforms. Additionally, snowball sampling, which is an effective method to engage hard-to-reach populations, will be employed [36]. We will be intentional in recruiting women of differing races, ethnicities, ages, and socioeconomic backgrounds by actively engaging with service providers and community-based agencies (e.g. anti-trafficking, housing) across Ontario that serve historically underrepresented and diverse communities. Once initial contact has been established, potential participants will be provided with a study flyer and an invitation letter detailing the study’s objectives, eligibility criteria, and the nature of their involvement. Further, they will be encouraged to disseminate the study information within their social circles to identify other individuals who meet the eligibility criteria.

### Interview guide

We will ask open-ended questions to elicit information about survivors’ experiences prior to, during, and after their trafficking situation, with particular attention paid to their health, well-being, and encounters with healthcare providers. Experiences of trafficking will be explored through questions such as, "Can you tell me how you first met your trafficker/s?" Experiences engaging with healthcare providers will be investigated through questions such as, “Can you tell me about your experiences accessing healthcare throughout your time of being trafficked?” The examination of the role of intersecting social identities in participants’ experiences will be explored through questions such as, “In what ways do you believe your race, gender, class, or migration status may have affected your experiences accessing care and interacting with providers?” Relationship dynamics within the trafficking experience will be captured through questions such as, “What was the relationship with the trafficker at the outset, and how did it shift over time?” Some items in the interview guide will be drawn or adapted from previous tools [19,30,51]. Mock interviews will be conducted among team members to pilot the interview guide, as recruiting for a pilot interview from what may already be a potentially small pool of willing sex-trafficked women may impact the final participant numbers.

### Sociodemographic questionnaire

The sociodemographic questionnaire will collect the following information: age, gender, gender identity, sexual orientation, disability status, racial identity/ethnic identity/cultural background, immigration status, area of residence (e.g. rural, urban, suburban), current living situation (e.g. living in transitional or temporary housing, living with a partner, living with family, living on your own with children, living on your own without children), highest level of education (e.g. elementary, high school, college, university), current work situation (e.g. unemployed looking for work, employed, self-employed), years on the job (if applicable), and approximate annual personal income (e.g. $0-$24,999,$25,000-$49,999).

### Data collection

Individual and semi-structured interviews will be held over Zoom. These interviews will be conducted by one research team member with extensive expertise working with sex-trafficked persons. The interviews will last approximately 90 minutes and will be audio or video-recorded. Before the interview begins, participants will be reminded that the interview is being recorded, but they can choose to keep their cameras turned off. Zoom will be used to generate transcripts from the interviews. Transcripts will be checked and re-checked against the recording for accuracy by the research team. Participants will choose/be assigned a pseudonym to be used on the transcript files. All information will be de-identified to maintain confidentiality. Each transcript and associated materials will be maintained in separate digital folders labelled with the participant’s pseudonym. These materials will be stored on a shared, secure OneDrive folder accessible only by the research team. Further, all audio and video recordings will be destroyed five years after the completion of any publications.

Questions to determine study eligibility will be emailed to all potential participants. Once eligibility has been confirmed, participants will receive a password via telephone for the password-protected Information and Consent Form and Sociodemographic Questionnaire. The Information and Consent Form will convey the goal and aims of the study, participation risks and benefits, steps to ensure confidentiality, and the intended use of the knowledge garnered from the study. Potential participants will be encouraged to participate in a consent discussion with a team member to (1) address any questions about their participation in the study and (2) provide sufficient time to review and understand the key elements of consent for the study prior to signing and sending back the form. Written consent will be obtained, after which point participants will complete and return the password-protected Sociodemographic Questionnaire. All participants will receive a comprehensive list of relevant support services. The list has been curated to include resources that do not require formal referrals and that provide services to diverse populations, including historically structurally marginalized groups (e.g. racial and sexual minorities, people with disabilities, and Indigenous persons). Further, acknowledging that participants may possess different levels of technological literacy, each participant will receive a manual that provides clear directions with images as well as text on how to use Zoom technology.

Upon receiving the digitally signed and completed Information and Consent Form and the Sociodemographic Questionnaire, an interview will be arranged at a time most suitable to the participants [52]. Before beginning the interview, we will ask participants if they are in a secure and private location. If not, the interview will not begin and will be rescheduled at a time of the participant’s choosing.

### Honorarium

Study participants will be compensated with a $75 gift e-card, emailed after completion of the interview. They will be asked to confirm via email when they have received the gift card.

### Data analysis

The transcribed interviews will be imported into Dedoose, a web-based qualitative data analysis software for coding [53]. Interview data will be analyzed deductively as well as inductively using Braun and Clarke’s [54] reflexive thematic analysis (RTA), grounded in intersectionality. RTA encourages thoughtful engagement with “reading and interpreting data to produce insights into your dataset that go beyond the obvious or surface-level content, and to noticing connections between the dataset and existing research, theory and the wider context” [55, p. 45]. Further, RTA calls on researchers to reflect critically on their role as researchers, research practices, and processes [55]. RTA is situated within a systematic framework for conducting qualitative research analyses that follow six phases: (1) familiarising yourself with your data, (2) generating initial codes, (3) searching for themes, (4) reviewing themes, (5) defining and naming themes, (6) producing the report. RTA encourages the use of one’s subjectivity throughout the research process, including drawing on ideas that emerge during team discussions, coding and analyzing the data, developing the final themes, and reporting results. Two research team members will initially independently code approximately four transcripts to establish the final codebook. Members of the CAG will provide interim and final feedback on the emerging themes and related quotes resulting from the data analysis.

We will apply intersectionality throughout the six phases of reflexive thematic analysis by taking into consideration participants’ identities (e.g. race, gender, sexual orientation), life circumstances (e.g. poverty, homelessness) as well as the various structures of power and oppression (e.g. racism, ableism, classism, and colonialism) that perpetuate inequities.

### Researchers’ positionality

The research team operates in a hospital and university setting and has varying professional experiences in women’s health, public health, social work, psychology, and gender-based violence. The team members possess expertise in qualitative research, have personal and professional experience relating to various forms of gender-based violence, and have training in trauma-informed and sensitive practices. We will actively practice reflexivity. This involves a deliberate effort by the researchers to scrutinize their own beliefs, assumptions, and practices and how these may unintentionally influence the study design and data analysis process [56]. To further support active reflexivity, we will use strategies such as challenging each other during regular team meetings when implicit assumptions, biases, or unsubstantiated opinions are expressed.

### Ethics and dissemination

This research was reviewed and approved by the Women’s College Hospital Research Ethics Board in September 2023 (REB #: 2023-0013-E) and the University of Toronto Research Ethics Board in October 2023 (Protocol #: 00045392). Written consent will be obtained from participants and stored in Women’s College Hospital’s secure One Drive folder. Recruitment of participants is expected to begin in March 2024. Findings will be disseminated in Open Access journals, freely available to diverse interdisciplinary audiences, including policymakers, health and social service providers, survivors of sex trafficking, and the public. Knowledge Translation tools, such as infographics, info cards, webinars, and a social media campaign, will be developed to share findings more broadly.

## Discussion

### Contributions

To our knowledge, the planned study will be the first of its kind in Ontario, Canada to apply intersectionality theory to explore the experiences of sex trafficking, health, well-being, intersecting oppressions, and interactions with healthcare from the perspectives of trafficked survivors. Given the lack of substantive research on sex trafficking in Ontario and its adverse impacts on women’s health, this study will lay the essential groundwork required to improve health services for sex trafficked survivors. The evidence generated may also hold the potential to enhance policies and practices for other sectors, including government, social services, and community.

### Limitations of study

We acknowledge that due to the qualitative nature of the study, the findings may not be generalizable to other jurisdictions. We also recognize that limiting recruitment to those able to read and speak in English may exclude some trafficked women from participating in our study. Further, the intention is to recruit survivors with varied social identities; however, those recruited may not fully represent Ontario’s diverse population. Despite these potential limitations, this study will make a significant contribution to the limited body of knowledge on domestic sex trafficking in Canada, enhancing the global understanding of the issue, particularly from a healthcare perspective.

## Supporting information

S1 Checklist COREQ (Consolidated criteria for Reporting Qualitative research) checklist. (PDF)
